# Application of the severe fever with thrombocytopenia syndrome prediction score: Differentiation of febrile diseases using basic laboratory parameters

**DOI:** 10.1371/journal.pone.0229920

**Published:** 2020-03-05

**Authors:** Euijin Chang, Jeong-Han Kim, Ji Hwan Bang, Wan Beom Park, Eu Suk Kim, Sang Won Park, Myoung-don Oh

**Affiliations:** 1 Department of Internal Medicine, Boramae Medical Center, Seoul, Republic of Korea; 2 Department of Internal Medicine, Seoul National University College of Medicine, Seoul, Republic of Korea; 3 Department of Internal Medicine, Seoul National University Bundang Hospital, Seongnam, Republic of Korea; Universite de Liege (B34), BELGIUM

## Abstract

**Background:**

The prolonged manifestation of concurrent leukopenia, thrombocytopenia and normal C-reactive protein (CRP) (named as SFTS prediction score) in febrile diseases is not usual and may be used to make an initial differential diagnosis, which is a characteristic finding of severe fever with thrombocytopenia syndrome (SFTS).

**Methods:**

The dynamics of SFTS prediction scores was investigated in SFTS patients. The study subjects for the comparison were febrile patients aged ≥ 16 years with SFTS scores of 2 (S2) or 3 (S3) who visited an emergency room for a 4-year study period. The dynamic distribution of S2 and S3 at presentation with regards to onset of illness, the characteristics of responsible diseases and the predictability of SFTS in both groups were described.

**Results:**

In 104 patients with SFTS, the daily proportion of S2 or S3 ranged from 58.3 to 100% from day (D) 1 to D12 after the onset of illness. The S2 subtype of ‘leukopenia plus thrombocytopenia’ and S3 represented 72.7–100% of all scores. In contrast, for the 130 patients in the febrile cohort, 73.8% of evaluations were distributed between D1 and D4 after the onset of illness, and 68.8% of patients had the S2 subtype of ‘leukopenia plus normal CRP’. Upper respiratory infection was the most frequent (50.0%) cause of diseases. Pneumonia (13.8%) and urosepsis (6.2%) initially presented with either S2 with normal CRP or S3 but had poor prognosis. The presence of S2 or S3 predicted SFTS with sensitivity and specificity of 0.85 (0.42–0.99; 95% CI) and 0.98 (0.98–0.98; 95% CI), respectively.

**Conclusion:**

The temporal distribution and composition of S2 or S3 were unique in several febrile diseases including SFTS, and the SFTS prediction score may be useful for differentiating febrile diseases in primary care settings of SFTS endemic areas.

## Introduction

Laboratory blood tests, such as complete blood count and chemistry, are useful basic tools for screening febrile diseases in primary care settings. Prolonged concurrent mixed presentation of leukopenia, thrombocytopenia and low or normal C-reactive protein (CRP) level is a characteristic finding in severe fever with thrombocytopenia syndrome (SFTS) [1, 2]. However, whether this phenomenon is unique to SFTS is not known. In our recent study, the SFTS prediction score was useful for differentiating SFTS from scrub typhus [3]. The SFTS prediction score ranged from 0 to 3 according to the concurrent presence of leukopenia (white blood cell count <4,000 /mm^3^), thrombocytopenia (platelet count <80,000 /mm^3^) and normal CRP (<1 mg/dL).

Changes in these laboratory parameters may be related to underlying comorbidities, the unique pathophysiology of diseases themselves or the net effects of the diseases such as sepsis. Fever is often a main sign of infectious diseases, but various noninfectious diseases also present with fever [4]. As most febrile diseases have accompanied inflammatory processes, leukocytosis or elevated CRP level are usually expected. As a result, concurrent presentation of leukopenia, thrombocytopenia, and normal CRP level may be unusual in patients with prolonged fever and, therefore, associated with differentiated conditions.

In this study, we analyzed the dynamics of these 3 parameters, leukopenia, thrombocytopenia and normal CRP level, alone or in combination in patients with SFTS. Then, we compared the dynamics of the parameters with those in another cohort of febrile patients visiting an emergency room to determine the distribution of the SFTS prediction score in this cohort. We intended to demonstrate that the SFTS scoring tool could effectively screen in SFTS patients and to characterize the other diseases having such unusual distribution of SFTS scores.

## Methods

### Dynamics of the SFTS prediction score in patients with SFTS

Serial temporal changes in 3 laboratory parameters, leukopenia (white blood cell count <4,000 /mm^3^), thrombocytopenia (platelet <80,000 /mm^3^) and normal CRP level (<1 mg/dL), were analyzed and plotted from the onset of illness to last follow-up in laboratory-confirmed SFTS patients. Illness meant any kind of clinical manifestations indicating the onset of current disease. Therefore, fever may be the first sign or one of the manifestations later in the clinical course. The SFTS prediction score was generated from the combination of those 3 parameters (1 point each for WBC count <4,000/mm^3^, platelet count <80,000/mm^3^ and CRP value <1 mg/dL; the total score ranged from 0 to 3). The SFTS prediction score was derived from our previous study [3]. There were 3 potential combination of subtypes for a score of 2: ‘leukopenia plus thrombocytopenia’, ‘leukopenia plus normal CRP’ or ‘thrombocytopenia plus normal CRP’.

The SFTS cases were retrospectively collected from 36 hospitals nationwide in South Korea from 2013 to 2015 [2]. Infection with the SFTS virus (SFTSV) was confirmed by detecting the M segment gene of SFTSV RNA using one-step reverse transcription polymerase reaction (RT-PCR), as described in a previous study [5]. The platelet count cut-off for thrombocytopenia was <80,000 /mm^3^, as determined using the mean value in SFTS cases in our previous study [2].

### Distribution of SFTS prediction scores in a cohort of febrile patients visiting an emergency room

We intended to apply the SFTS prediction scores into an individual community-based tertiary hospital level and to find out how it was working for screening SFTS patients. All febrile patients visiting the emergency room (ER) were eligible for the study. Specifically, the inclusion criteria for an initial screening were febrile patients (≥37.8 °C) aged ≥16 years old visiting ER of the study hospital in the past 4 years (2015–2018). If the patients visited the ER multiple times during the study period, only the initial visit was included in the study. Then, among the initially screened patients, only those who had an SFTS prediction score of 2 (S2) or 3 (S3) were included in the clinical and laboratory analyses. Clinical variables included underlying comorbidities, onset of illness, onset of fever, date of initial visit, duration of hospitalization, in-hospital mortality and final diagnosis of fever, which were reviewed using electronic medical records. Laboratory variables included complete blood count and CRP. The worst values for the parameters within 24 hours of the initial visit were used. It was a routine practice at ER to do basic laboratory tests (complete blood count, chemistry, CRP, urinalysis, simple x-ray, blood and urine cultures) for the initial evaluation of febrile patients except simple cases.

**Fig 1:**
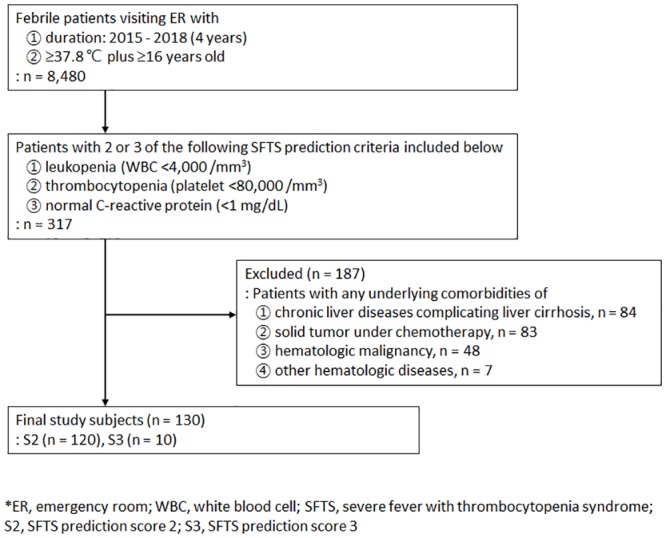
The study design.

Finally, patients with common accountable comorbidities (chronic liver diseases, solid tumors under chemotherapy, hematologic malignancy and other hematologic diseases) for leukopenia and thrombocytopenia were excluded. Chronic liver diseases included liver cirrhosis complicated by chronic viral or nonviral hepatitis. Only those without common comorbidities contributing to S2 or S3 were subjected to the final analysis, as depicted in Fig 1.

The distribution of the initial S2 or S3 was analyzed and plotted by onset of illness or fever. The causative diagnoses for the fever associated with S2 or S3 in specific time periods were compared. The study hospital, Boramae Medical Center, is a 767-bed university-affiliated teaching hospital with one ER, three intensive care units and 17 general wards located in a metropolitan city. It serves neighboring communities and patients referred from remote rural areas. By the institutional rule for patient care the final diagnosis was guided and confirmed by expert physicians in charge. The hospital has an active consultation system for infectious diseases and febrile diseases with unknown diagnosis are consulted to infectious diseases (ID) physicians. All the SFTS patients were also consulted to ID physicians and led to the final diagnosis.

### Statistical analysis

Chi-square or Fisher’s exact tests were used to analyze the categorical variables. T-tests was used to compare the continuous variables. Multivariate regression analysis was performed for the age-adjusted risk analysis for fatality in SFTS group. P <0.05 was considered to have statistical significance. Sensitivity, specificity, positive likelihood ratio and negative likelihood ratio with a 95% CI for the detection of SFTS in febrile patients were calculated using SFTS prediction scores of S2 or S3 (SPSS v 20.0, Armonk, NY: IBM Corp.).

### Ethics approval and consent to participate

This study was approved by the institutional review board of Boramae Medical Center (30-2018-80), which waived the need to obtain consent from the patients. Personal information was deidentified before data retrieval, and the anonymized data were processed by different analyzers. All clinical investigations were conducted according to the principles expressed in the Declaration of Helsinki.

## Results

### Dynamics of leukopenia, thrombocytopenia and normal CRP in SFTS patients

**Fig 2:**
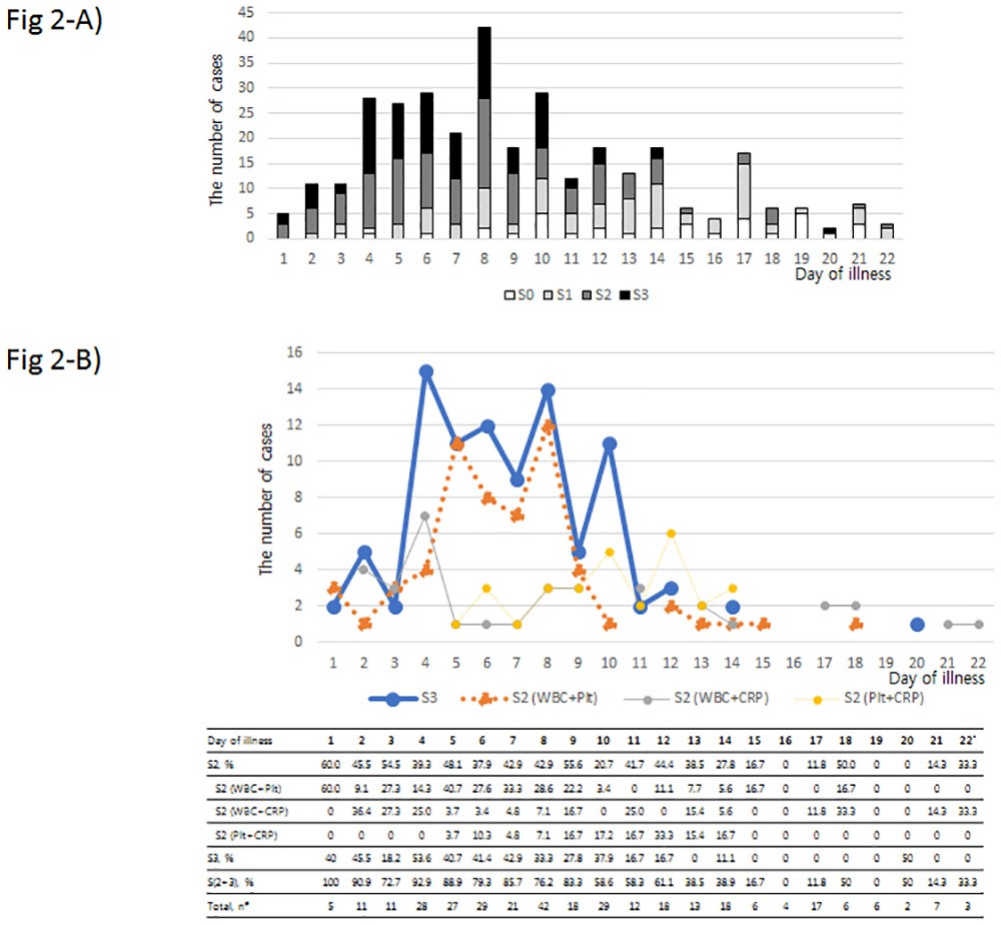
The temporal dynamics of SFTS prediction scores in patients with SFTS (n = 104). **A)** Daily proportion of SFTS prediction score ranging from 0 (S0) to 3 (S3). **B)** Temporal changes in S3 and the 3 subtypes of S2. (#) ‘Total, n’ indicates the number of patients with the laboratory tests on the given day. (*) During D23-D51, there were 32 more laboratory follow-ups, and only 2 of them were classified as S2. The small distribution after D22 is not shown. SFTS = Severe fever with thrombocytopenia syndrome; WBC = leukopenia (white blood cell count <4,000/mm^3^); Plt = thrombocytopenia (platelet <80,000 /mm^3^); CRP = normal level of CRP (<1 mg/dL).

Among all 172 patients who were diagnosed with SFTSV infection in South Korea during the study period, 104 patients who had appropriate information were subjected to a final analysis. Male sex was 52.9% (55/104) and average age was 67.4 years (61.0–78.2 years, interquartile range [IQR]). The case fatality rate was 39.4% (41/104). The serial temporal changes in leukopenia, thrombocytopenia and normal CRP level were followed in reference to the onset of illness (Fig 2). The serial temporal distribution of S2 or S3 spanned approximately 2 weeks after the onset of illness in SFTS patients. The daily proportion of S2 or S3 between D1 and D12 ranged from 58.3 to 100%. The S2 subtype ‘leukopenia plus thrombocytopenia’ and S3 were the main components, comprising 72.7–100% of all scores (S0—S3) from D1 to D9. In case of S2, ‘leukopenia plus thrombocytopenia’ or ‘leukopenia plus normal CRP’ subtypes were the majority (39.3%–60.0%) from D1 to D4, but ‘leukopenia plus thrombocytopenia’ was dominant from D5 to D9. ‘Thrombocytopenia plus normal CRP’ was zero from D1 to D4 and then gradually increased and peaked at D12, reaching zero again after D14. After D14, few patients were classified as S2 or S3.

**Fig 3:**
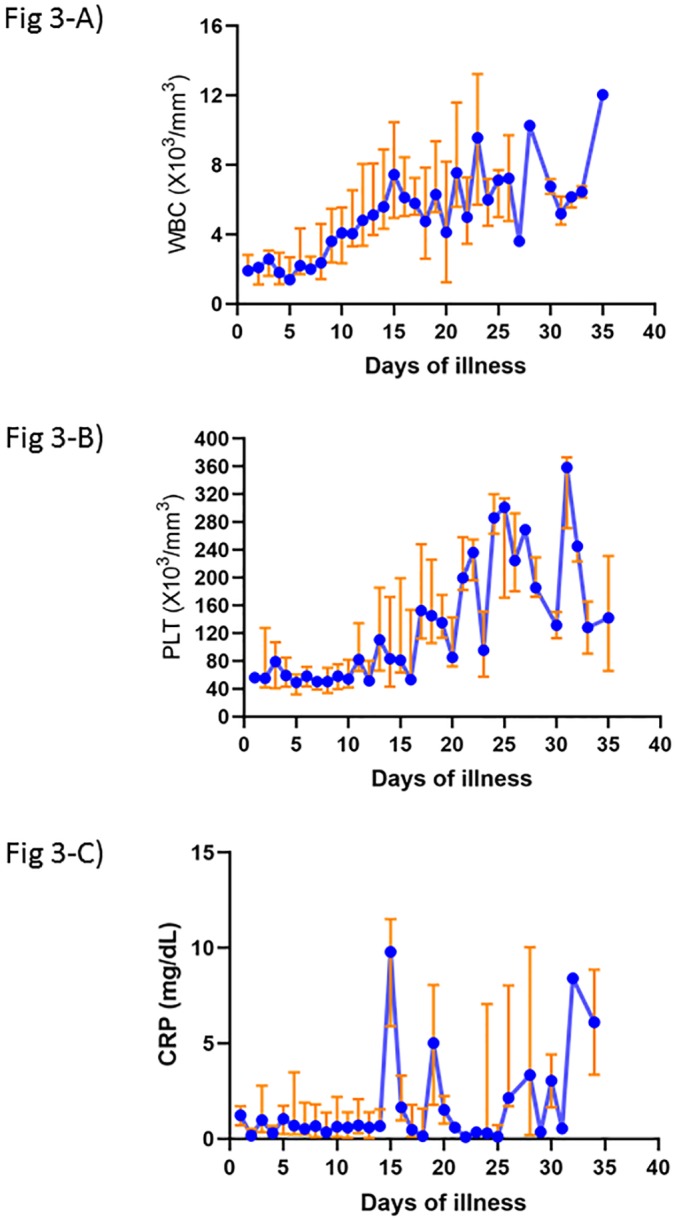
Changes in laboratory parameters over time in 104 patients with severe fever with thrombocytopenia syndrome. **A)** Change of white blood cell. **B)** Change of platelet. **C)** Change of C-reactive protein. The Y-axis denote the median (blue line) and interquartile (red line) values. WBC, white blood cell; PLT, platelet; CRP, C-reactive protein.

The absolute value and time trends of WBC, platelet and CRP in the SFTS patients from the onset of illness were presented (Fig 3). Leukopenia, thrombocytopenia and low CRP value are prominent features from the beginning of illness, and these are persistent until second week.

For additional reference, we analyzed the distribution of SFTS scores at initial visit and divided them into non-fatal and fatal groups (Table 1). The SFTS patients initially visited the hospital around median 5th day of illness and majority of them presented with S2 or S3 (96/104, 92.3%). There were significant differences in the distribution of SFTS scores at the initial visit between the two groups in a univariate analysis. Fatal group had higher rates of ‘leukopenia + thrombocytopenia’ (P <0.001) or ‘thrombocytopenia + increased CRP’ (P = 0.014). The fatal group also had a higher rate of ‘leukopenia + thrombocytopenia + increased CRP’ (P = 0.044). In an age-adjusted multivariate analysis, however, only S2 of ‘leukopenia + thrombocytopenia’ had a tendency of fatality (P = 0.054). Old age itself was a constant risk factor for fatality (P = 0.003; OR 1.072, 95% CI 1.024–1.121) (Table 1).

**Table 1:** The distribution of SFTS prediction scores (S) at initial visit regardless of the onset of illness among patients with severe fever with thrombocytopenia syndrome (N = 104).

SFTS scores		Day of illness at presentation*	Total (%)	Non-fatal N = 63	Fatal N = 41	Univariate P value	Multivariate^#^ P value (OR, 95% CI)
S0		6	1 (1.0)	1	0	-	-
S1		4 (3–6)	7 (6.7)	5	2	0.701	-
	WBC			2	1	-	-
	PLT			2	1	-	-
	CRP			1	0	-	-
S2		5 (4–6)	53 (51.0)	26	27	0.014	-
	WBC+PLT			10	20	<0.001	0.054 (0.964–46.556)
	WBC+CRP			4	6	0.187	0.558 (0.025–7.345)
	PLT+CRP			12	1	0.014	0.722 (0.188–11.141)
S3		5 (4–8)	43 (41.3)	31	12	0.044	0.798 (0.121–5.073)

Statistics:

*median (interquartile range).

^#^age-adjusted multivariate analysis for fatality.

WBC, leukopenia (<4,000 /mm^3^); PLT, thrombocytopenia (<80,000 /mm^3^); CRP, normal C-reactive protein (<1 mg/dL); OR, odds ratio.

### Distribution of leukopenia, thrombocytopenia and normal CRP in a cohort of febrile patients visiting an ER

During the study period of 4 years, 8,480 patients meeting the inclusion criteria of fever (≥37.8 °C) and aged ≥16 years visited the ER. Among them, 317 patients had S2 (277) or S3 (40). Out of these 317 patients, 187 patients had comorbidities causing cytopenia (84 with chronic liver diseases, 83 with solid tumor under active chemotherapy, 48 with hematologic malignancies and 7 with other hematologic diseases). Therefore, the 130 patients (120 with S2 and 10 with S3) were subjected to final analysis and comparison (Fig 1). The yearly proportion of the study subjects was similar (P = 0.380). Male sex was 43.8% (57/130) and average age was 52.1 years (32.3–68.0, IQR). There was no difference in sex (P = 0.600), but the average age was younger than that of the SFTS patients (P <0.001).

**Fig 4:**
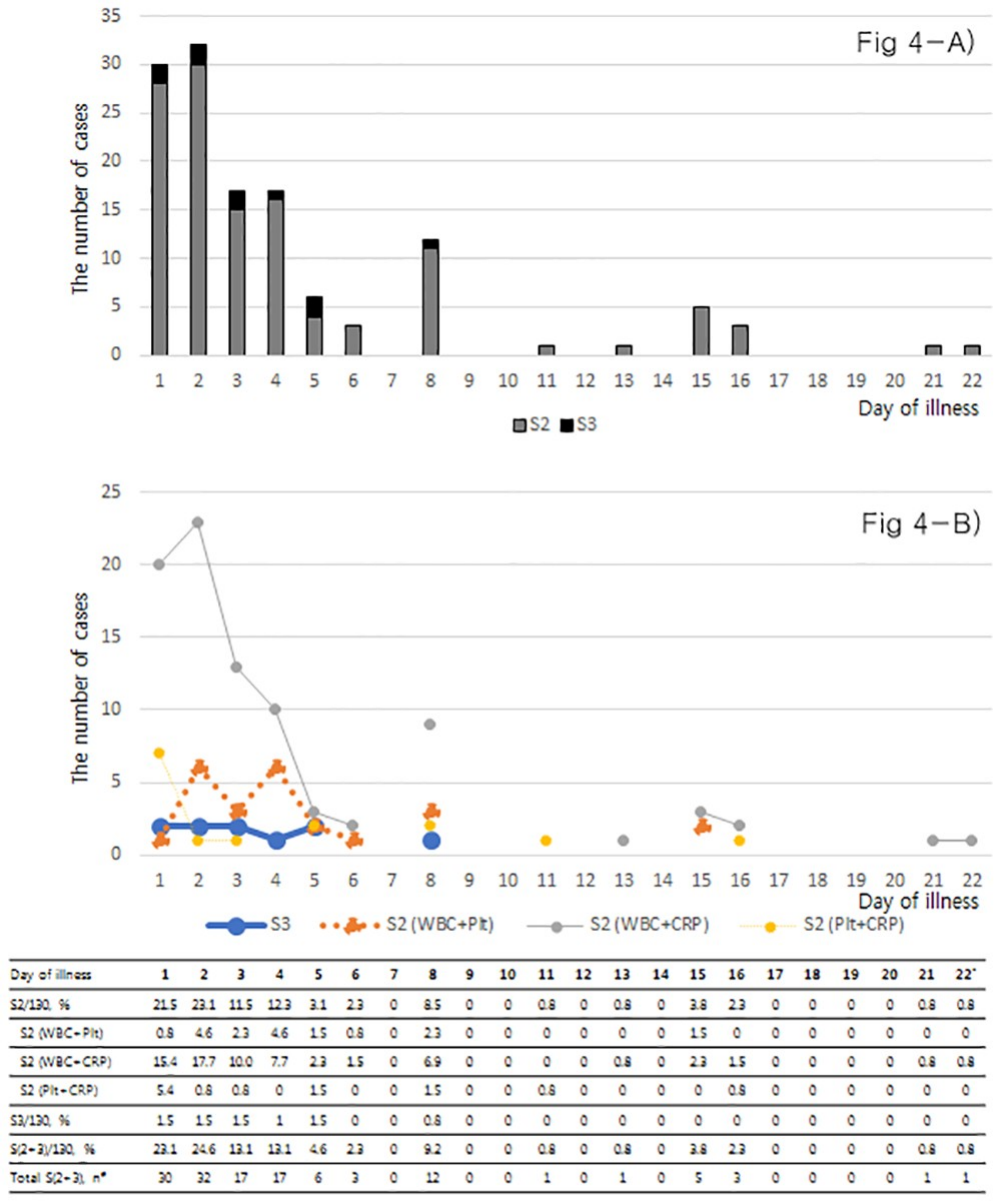
The distribution of initial SFTS prediction score 2 (S2) or 3 (S3) at presentation in febrile patients in an emergency room (n = 130). **A)** Daily proportion of S2 and S3. **B)** The distribution of S3 and the 3 subtypes of S2. (#) ‘Total, n’ indicates the number of patients who visited the emergency room for the first time on the given day. (*) After D22, there was only one patient, who was evaluated on D32. The small distribution after D22 is not shown. SFTS = Severe fever with thrombocytopenia syndrome; WBC = leukopenia (white blood cell count <4,000/mm^3^); Plt = thrombocytopenia (platelet <80,000 /mm^3^); CRP = normal level of CRP (<1 mg/dL).

The febrile patients visited the ER from D1 to D32 after the onset of illness (Fig 4). Most of the patients (117/130, 90.0%) with S2 or S3 visited the ER from D1 to D8. The period of D1 to D4 was the most frequently visited, which included 73.8% (96/130) of patients; the S2 subtype of ‘leukopenia plus normal CRP’ was the main presentation, including 68.8% (66/96) of patients. The proportion of S3 composed only 7.7% (10/130) of patients. The major causes of fever presenting with S2 or S3 were upper respiratory infection (50.0%, 65/130), pneumonia (13.8%, 18/130), urosepsis (6.2%, 8/130), acute gastroenteritis (4.6%, 6/130), SFTS (4.6%, 6/130) and drug fever (4.6%, 6/130) (Table 2). The S2 subtype of ‘leukopenia plus normal CRP’ was more common with upper respiratory infection and acute gastroenteritis than were the other S2 subtypes of ‘leukopenia plus thrombocytopenia’ or ‘thrombocytopenia plus normal CRP’ (P <0.001). The causative diseases in the 10 patients with S3 were SFTS (4 cases), pneumonia (3), drug fever (2) and heat stroke (1). There was no fatality in the upper respiratory infection, but high fatality was observed in pneumonia (27.8%), urosepsis (12.5%), SFTS (50%) and heat stroke (33.3%).

**Table 2:** Characteristics of the febrile patients with S2 or S3 at presentation without comorbidities causing cytopenia (N = 130).

	D1–D4				D5–D6				D8				D11–D32				
Diagnosis	N	D_fever (day)	D_admission (day)	Death (%)	N	D_fever (day)	D_admission (day)	Death (%)	N	D_fever (day)	D_admission (day)	Death (%)	N	D_fever (day)	D_admission (day)	Death (%)	Total N
Upper respiratory infection	49	2 (1–8)	1 (1–79)	0	3	5 (4–6)	1 (1–1)	0.0	8	8 (1–8)	1 (1–2)	0.0	5	1 (1–2)	1 (1–1)	0.0	65
Pneumonia	14	1 (1–4)	11 (1–135)	21.4	1	3	2	100					3	15 (1–15)	8 (2–14)	33.3	18
Urosepsis	8	1 (1–3)	10 (1–36)	12.5													8
Acute gastroenteritis	5	1 (1–4)	1 (1–6)	0.0									1	1	1	0.0	6
SFTS	2	3.5 (3–4)	10 (6–14)	50.0	1	5	5	100	2	8 (8–8)	9 (7–11)	50.0	1	13	6	0.0	6
Drug fever	2	3.5 (1–6)	9 (7–11)	0.0	1	1	1	0.0	1	1	66	0.0					4
Intraabdominal infection	2	1.5 (1–2)	4.5 (2–7)	0.0	1	5	22	0.0									3
Heat stroke	3	1 (1–1)	1 (1–2)	33.3													3
Malaria, vivax													1	15	6	0.0	1
Adrenal insufficiency	1	1	5	0.0													1
Dengue	1	4	8	0.0													1
Acute viral hepatitis	1	2	10	0.0													1
Acute HIV syndrome									1	8	1	0					1
Undetermined	8	1 (1–3)	2 (1–9)	0.0	2	6 (6–6)	1 (1–1)	0.0					2	6.0 (1–11)	6.5 (5–8)	0.0	12
	96				9				12				12				130

Statistics: The unit of values is median (range) for D_fever and D_admission. If the number of patients is only one, a single value is shown.

D, day of illness (D1 means 1^st^ day of illness onset); D_fever, duration of fever at presentation (day); D_admission, duration of hospitalization (day); Death, in-hospital mortality (%).

SFTS, severe fever with thrombocytopenia syndrome; HIV, human immunodeficiency virus.

Among the 65 cases of upper respiratory infection, 3 cases were influenza, but the others were nondiagnostic mainly because specific diagnostic tests were not performed. The number of cases after D4 was relatively small, and they needed only a short period of hospitalization (average 1–1.8 days). Among the 18 cases of pneumonia, 13 cases were bacterial infection, 4 were suggestive of viral infection and 1 was tuberculosis. The proportion of cases from D1 to D4 was 77.8%, but the average duration of hospitalization was long (21.8 days). Among the 8 cases of urosepsis, 7 cases were accompanied by bacteremia. All cases of urosepsis presented within D1 to D4, but the average duration of hospitalization was also long (12.4 days). All cases of acute gastroenteritis lacked etiologic diagnosis. The proportion of patients hospitalized after D7 was 19.2% (25/130), and the causative diseases were upper respiratory infection (13 cases), pneumonia (3), SFTS (3), drug fever (1), vivax malaria (1), acute HIV syndrome (1) and undetermined etiology (2).

During the study period, 7 SFTS patients were reported in the study hospital and 6 of them were screened in with S2 or S3. The other one case was excluded because only leukopenia was present on the initial visit day (WBC 1,880/mm^3^, platelet 110,000/mm^3^, CRP 2.73 mg/dL) which was 4^th^ day of illness. The patient visited the ER again on 6^th^ day and reached S2 (WBC 2,120/mm^3^, platelet 69,000/mm^3^, CRP 3.45 mg/dL), and reached S3 on 10^th^ day (WBC 2,800/mm^3^, platelet 44,000/mm^3^, CRP 0.87 mg/dL). The 6 SFTS patients included in the study demonstrated an even distribution of scores over the 2-week duration (Table 2). Regarding the 6 SFTS patients, male sex was 33.3% and median age was 68 years (range, 57–89). Chief complaints at the first visit were fever (3/6), altered mentality (2/6) and hematuria (1/6). Underlying comorbidities except those associated with cytopenia were hypertension (4/6), diabetes mellitus (2/6), atrial fibrillation (1/6) and hypothyroidism (1/6). Median values of white blood cell, platelet and CRP were 1,765/mm^3^ (range, 670–3,510), 35,300/mm^3^ (range, 7,000–89,000) and 0.44 mg/dL (range, 0.01–1.55), respectively.

### Prediction of SFTS in febrile patients using the SFTS prediction score

The sensitivity, specificity, positive likelihood ratio and negative likelihood ratio were calculated for the 6 SFTS patients screened in by S2 or S3 as to the total 7 SFTS patients reported during the study period and all 8,480 study subjects. For the study subjects screened as S2 or S3 (n = 317), the performance were sensitivity of 0.85 (95% CI, 0.42–0.99), specificity of 0.96 (0.96–0.96), positive likelihood ratio (LR) of 23.35 (11.35–27.12), negative likelihood ratio (LR) of 0.14 (0.01–0.60), positive predictive value (PPV) of 0.02 (0.01–0.02) and negative predictive value (NPV) of 1.00 (1.00–1.00). In case of the S2 or S3 subjects with further exclusion of accountable comorbidities for cytopenia (n = 130), the performance were sensitivity of 0.85 (0.42–0.99), specificity of 0.98 (0.98–0.98), positive LR of 58.56 (28.16–68.34), negative LR of 0.14 (0.01–0.58), PPV of 0.02 (0.01–0.02) and NPV of 1.00 (1.00–1.00).

## Discussion

Leukopenia, thrombocytopenia, and low CRP level are well known manifestations in SFTS [1, 2, 6], but it is not known how these parameters interact and what is their relationship with other febrile diseases. In our previous study, we presented the individual dynamics of these 3 parameters in SFTS [2]. In this current study, we showed that for patients meeting the criteria for SFTS predictive scores of S2 or S3, the components of leukopenia, thrombocytopenia or normal CRP in SFTS were distributed up to approximately 2 weeks from the onset of illness and were present in 58.3% to 100% of patients on any given day. There were changes in dominance over daily intervals, but the S2 subtype of ‘leukopenia plus thrombocytopenia’ and S3 were the major combinations in SFTS. In the febrile cohort, in contrast, patients presenting with S3 were rare (7.7%). Among the febrile cases presenting with S2, the predominant subtype was ‘leukopenia plus low CRP’ (68.8%), and the temporal distribution of S2 and S3 was narrow, mostly distributed within day 4 (D4).

Upper respiratory infection of probable viral origin was the most common cause of illness in the febrile cohort, and the prognosis was excellent. The patients required, if any, hospitalization for only 1 or 2 days, and there was no mortality. Definite diagnostic tests were not performed in most of cases with the upper respiratory infections due to the mild clinical presentation. Interestingly, there were patients with pneumonia and urosepsis of bacterial origin presenting with S3 or S2 having normal CRP and a short febrile period (<2 days). The duration of hospitalization, however, was long and the mortality was high (Table 2). Normal CRP levels changed to high values in a few days. As severe bacterial pneumonia and urosepsis in a hyperacute stage may present with cytopenia and normal CRP, normal CRP in an acute presentation should be cautiously interpreted. Other febrile diseases with intrinsic features of cytopenia and normal CRP included SFTS, drug fever, heat stroke, dengue, acute viral hepatitis, vivax malaria and acute HIV infection. Among them, SFTS and drug fever deserve particular notice because they were steadily observed over 2 weeks and 1 week, respectively.

In our febrile cohort from a single hospital, the sensitivity and specificity of the SFTS prediction scores of S2 or S3 for SFTS were 0.85 (0.42–0.99, 95% CI) and 0.96 (0.96–0.96, 95% CI), respectively. As the clinical decision is based on multifaceted evidence, the predictive power may be enhanced if the prediction tool is interpreted together with other clinical clues for the individual diseases [6]. If the suspicion of SFTS is equivocal, follow-up laboratory tests or clinical examination up to 1 week from the onset of illness may be helpful to consolidate the clinical suspicion. As shown in Fig 2, the number of 1st visit and the presentation of S2 or S3 peaked around week 1. To apply the SFTS prediction tool in the practice, appropriate estimation of the onset of illness is essential to gauge its applicable time point.

Although there were 108 cases of scrub typhus reported at ER during the study period, any case of scrub typhus was not included in the study because they all had score 0 or 1. As we originally introduced the SFTS prediction score to differentiate SFTS from scrub typhus [3], we could clearly differentiate scrub typhus using this prediction tool. The sensitivity and specificity of this tool to differentiate these two diseases in our practice was 0.85 (0.50–0.85, 95% CI) and 1.00 (0.99–1.00, 95% CI), respectively. The list of diseases to be differentiated may differ according to the regional epidemiology of febrile diseases.

As the diagnostic confirmation was done by RT-PCR of serum, the SFTS patients in the study were all in a viremic phase. This finding might raise the possibility of selection bias for SFTS subjects with more severe presentation [1]. However, milder cases which can be serologically diagnosed may not be clinically challenging. We excluded the patients with comorbidities that cause cytopenia such as chronic liver diseases and malignancies under immunosuppressive therapy. Such comorbidities themselves would significantly lead to bias by confusing the real cause of leukopenia or thrombocytopenia. The exclusion reduced the total number of study subjects but might help describe the causative diseases more clearly. This approach may omit SFTS or other diseases with S2 or S3 combined with other comorbidities, but the frequency may not be significant, as the patients with such comorbidities may be less frequently exposed to risky outdoor activities. In our previous study with SFTS (n = 120), such comorbidities were rare, and in the current study, no SFTS patient was excluded by these comorbidities [3].

CRP is an acute phase reactant that interacts with bacterial lipopolysaccharide [7] and mainly synthesized by hepatocytes [8]. Interleukin-6 is a main regulator of CRP synthesis and secreted from macrophages upon stimulation by bacterial lipopolysaccharides [9]. Viral pathogens have no lipopolysaccharide. Elevated levels of CRP usually indicate bacterial infection. Bone marrow suppression and peripheral sequestration/destruction of platelets in the spleen were considered the main causes of cytopenia in SFTS [10, 11]. Dengue typically presents with thrombocytopenia and leukopenia [12]. Low CRP (< 1.0 mg/dl) is usual in dengue, but elevated CRP (> 3.0 mg/dl) level is found in severe dengue cases [13]. Thrombocytopenia and leukopenia are common findings in malaria as well, accounting for 50–70% and 10% of cases, respectively [14, 15]. The CRP level usually ranged from 1 to 2 mg/dl [16].

This study has several limitations to consider. First, we analyzed the febrile cohort from a single institution in one country. Although many patients were enrolled over the 4-year duration, various diseases of specific endemicity or institutional features might not be included. However, most common diseases encountered in primary care settings were included, and the clinicians can interpret such specific diseases based on their own regional epidemiology. Second, due to the low prevalence of SFTS, PPV was very low (0.02; 95% CI, 0.01–0.02) in contrast to high NPV (1.00; 1.00–1.00). As a multifaceted assessment is needed to make an optimal clinical decision, this simple tool may not be enough to address such a complex process. We have suggested, however, the simplest approach to screen febrile diseases using common and practical laboratory parameters that can be applied in usual primary care settings. Other ancillary methods or clinical clues will further help the diagnostic process.

## Conclusions

We showed the dynamics of the SFTS prediction scores in SFTS patients in reference to the onset of illness, then compared the dynamics in a febrile cohort at ER. The temporal distribution and composition of S2 or S3 were unique in several febrile diseases including SFTS. This tool may be used as an initial screening method of SFTS among febrile patients in a low prevalence area, and may be particularly useful to primary care physicians who are not familiar with SFTS. And it can be incorporated into electronic medical recording system to generate automatic alarms to indicate an urgent differential diagnosis. However, as the accuracy of the tool is low, it must be combined with further clinical diagnostics or consultation to guide a final diagnosis. The dynamics of SFTS scores helped understand the unique presentation of several febrile diseases.
